# Characterization of SOX2, OCT4 and NANOG in Ovarian Cancer Tumor-Initiating Cells

**DOI:** 10.3390/cancers13020262

**Published:** 2021-01-12

**Authors:** Mikella Robinson, Samuel F. Gilbert, Jennifer A. Waters, Omar Lujano-Olazaba, Jacqueline Lara, Logan J. Alexander, Samuel E. Green, Gregory A. Burkeen, Omid Patrus, Zinia Sarwar, Ryne Holmberg, Christine Wang, Carrie D. House

**Affiliations:** 1 Biology Department, San Diego State University, San Diego, CA 92106, USA; mrobinson3@sdsu.edu (M.R.); sfgilbert@sdsu.edu (S.F.G.); jwaters5055@sdsu.edu (J.A.W.); olujano3108@sdsu.edu (O.L.-O.); jlara4692@sdsu.edu (J.L.); loganalexander6@yahoo.com (L.J.A.); seg30green69@gmail.com (S.E.G.); gburkeen@sdsu.edu (G.A.B.); omid.patrus@westernu.edu (O.P.); Zsarwar@evofem.com (Z.S.); rholmberg@sdsu.edu (R.H.); clwang@sdsu.edu (C.W.); 2 Moores Cancer Center, University of California San Diego, La Jolla, CA 92037, USA

**Keywords:** ovarian cancer, tumor-initiating cells, chemotherapy resistance, SOX2, OCT4, NANOG, spheroids

## Abstract

**Simple Summary:**

SOX2, OCT4, and NANOG are enriched in ovarian cancer spheroids and correlate with tumor-initiating cell markers, CD117 and ALDH/CD133. SOX2, relative to OCT4 or NANOG, is a stronger indictor of chemoresistance, tumor-initiation, and recurrent disease. Including SOX2 in evaluation of ovarian cancer TICs may improve reliability of TIC markers and our understanding of mechanisms of relapse.

**Abstract:**

The identification of tumor-initiating cells (TICs) has traditionally relied on surface markers including CD133, CD44, CD117, and the aldehyde dehydrogenase (ALDH) enzyme, which have diverse expression across samples. A more reliable indication of TICs may include the expression of embryonic transcription factors that support long-term self-renewal, multipotency, and quiescence. We hypothesize that SOX2, OCT4, and NANOG will be enriched in ovarian TICs and may indicate TICs with high relapse potential. We evaluated a panel of eight ovarian cancer cell lines grown in standard 2-D culture or in spheroid-enriching 3-D culture, and correlated expression with growth characteristics, TIC marker expression, and chemotherapy resistance. RNA-sequencing showed that cell cycle regulation pathways involving SOX2 were elevated in 3-D conditions. HGSOC lines had longer doubling-times, greater chemoresistance, and significantly increased expression of SOX2, OCT4, and NANOG in 3-D conditions. CD117+ or ALDH+/CD133+ cells had increased SOX2, OCT4, and NANOG expression. Limiting dilution in in vivo experiments implicated SOX2, but not OCT4 or NANOG, with early tumor-initiation. An analysis of patient data suggested a stronger role for SOX2, relative to OCT4 or NANOG, for tumor relapse potential. Overall, our findings suggest that SOX2 may be a more consistent indicator of ovarian TICs that contribute to tumor repopulation following chemotherapy. Future studies evaluating SOX2 in TIC biology will increase our understanding of the mechanisms that drive ovarian cancer relapse.

## 1. Introduction

High Grade Serous Ovarian Cancer (HGSOC) is the most lethal gynecological malignancy in women. Although most patients respond to platinum-based chemotherapies, over 70% relapse with therapy-resistant disease within 18 months [1]. Ovarian cancer recurrence may involve a small population of tumor-initiating cells (TICs) that can resist chemotherapy and efficiently repopulate tumors. TICs share properties of tissue stem cells such as quiescence, asymmetric division, and long-term self-renewal [2,3,4,5,6]. Consequently, chemotherapeutic drugs are inefficient at eliminating TICs, and these cells likely drive recurrence [7,8].

The identification of ovarian TICs has traditionally relied on a combination of cell surface proteins, mainly CD133, CD44, and CD117, as well as the activity of aldehyde dehydrogenase (ALDH) enzymes [9,10,11,12]. Ovarian cancer cells have heterogeneous expression of TIC markers that are often cell-line or patient specific. Previous studies showed that SOX2 [13] or ALDH [14], CD133, and CD117 [10] gene expression levels varied across cell lines and patient samples. The genetic heterogeneity of ovarian cancer, as well as the disease stage and/or tissue origin for subsets of ovarian TICs, contribute to the expression of different markers [7,15,16].

Given their role in maintaining pluripotency and long-term self-renewal, the embryonic transcription factors SOX2, OCT4, and NANOG are recognized as part of the stem cell signature [13,17]. Indeed, enhanced expression of SOX2, OCT4, and/or NANOG are typical of ovarian TICs, and their expression enhances spheroid formation, drug efflux, and chemoresistance [9,12,18,19,20,21]. However, to our knowledge, to date, no comprehensive study has characterized SOX2, OCT4, and NANOG in the most commonly used ovarian cancer cell lines or determined whether their expression correlates with specific surface markers and other features of TICs.

To address this deficiency, we investigated SOX2, OCT4, and NANOG expression in a panel of ovarian cancer cell lines to identify correlations with growth properties, spheroid-forming potential, traditional TIC marker expression, tumorigenesis, and chemoresistance. Cells were cultured as spheroids in 3-D conditions and compared to standard growth in 2-D conditions. We showed that SOX2, OCT4 and NANOG are consistently enriched in HGSOC lines cultured in 3-D, and that their expression correlates with genes encoding traditional TIC markers. Cells expressing CD117 or CD133 in combination with ALDH activity identify cells with relatively high SOX2, OCT4, and NANOG expression. Cells with enhanced tumor-initiation capacity and resistance to chemotherapy in vitro have significantly enriched SOX2, but not OCT4 or NANOG. These data provide the first comprehensive evaluation of SOX2, OCT4 and NANOG in TICs from commonly used ovarian cancer cell lines, and suggest that SOX2 may be a more reliable marker to identify TICs with relapse potential.

## 2. Materials and Methods

### 2.1. Cell Lines and Culture Conditions

Cells were maintained as recommended by manufacturer or cell line source (Table S1). ACI23 cell line was maintained as described previously [21]. All 2-D monolayer cultures (2-D) were maintained in standard media with 10% FBS in tissue-culture treated flasks. Except where noted, 3-D cultures (3-D) were maintained in stem cell media: DMEM:F12 supplemented with 1% KnockOut serum replacement, 0.4% bovine serum albumin, and 0.1% insulin-transferrin-selenium and cultured in non-treated tissue culture flasks. 3-D cultures were supplemented with human recombinant epidermal growth factor (EGF) and basic fibroblast growth factor (FGF) every 2–3 days for final concentration of 20 ng/mL and 10 ng/mL, respectively.

### 2.2. Spheroid Formation Assay

To generate spheroids, 500 cells/well in 100 μL were seeded in ultralow attachment, flat bottom 96-well plates in either 3-D or 2-D media. After 4–7 days in culture, whole wells were stained with Hoechst and imaged at 10× magnification using the ImageXpress Pico. Spheroids ranging in size from 30–600 μm were counted and analyzed using the CellReporterXpress software. Spheroid efficiency is defined as number of spheroids/500 cells per well.

### 2.3. siRNA Transfections and Western Blots

Cells were plated in a six-well plate at a density of 4 × 10^5^ cells per well and cultured overnight. The following day, 30nM pools of 4 siRNAs targeting either SOX2 or a non-targeting control (Dharmacon, Lafayette, CO, USA) was transfected using 7.5 μL Lipofectamine RNAiMAX (ThermoFisher Scientific, Waltham, MA, USA) according to the manufacturer’s instructions. Cells were allowed to incubate for a 24-h period before being collected for downstream experiments. Cells were replated and cultured for an additional 48 h for SOX2 knockdown validation.

Whole cell protein was extracted using standard methods with RIPA lysis buffer supplemented with Halt Protease and Phosphatase Inhibitor Single-Use Cocktail (ThermoFisher Scientific, Waltham, MA, USA). Pierce Rapid Gold BCA Protein Assay Kit was used for protein quantitation. SDS-PAGE was performed using NuPAGE 4-12% Bis-Tris Protein Gels and NuPAGE MOPS SDS Running Buffer. Proteins were transferred to a PVDF Transfer Membrane, blocked with 5% milk, and incubated overnight at 4 °C with primary antibodies from Cell Signaling Technology (Danvers, MA, USA) or MilliporeSigma (Burlington, MA, USA) (Table S2). Secondary antibody was purchased from Cell Signaling Technology (Danvers, MA, USA) and incubation was carried out using a 1:2000 dilution at room temperature for 1 h. SuperSignal Chemiluminescent Substrate was used for protein detection in the iBright CL1000 Imaging System (ThermoFisher Scientific, Waltham, MA, USA). Invitrogen iBright Analysis Software (ThermoFisher Scientific, Waltham, MA, USA) was used to determine relative protein expression of SOX2.

### 2.4. RNA Extraction and Quantitative Reverse Transcription PCR (qRT-PCR)

Total RNA was isolated using either NucleoSpin RNA Plus purification kit or, in the case of FACS-sorted cells, Direct-zol RNA Miniprep Plus kit per manufacturer’s instructions. Final RNA concentration was determined with a SpectraMax QuickDrop and converted into cDNA using the High-Capacity cDNA Reverse Transcription Kit. Quantitation and normalization of gene expression was performed with Taqman Fast Advanced Master Mix and Taqman probe assays (ThermoFisher Scientific, Waltham, MA, USA) with GAPDH as a control using the comparative threshold cycle method. TaqMan product information provided in Table S2. Reactions were run and analyzed with the QuantStudio 3 machine and Design software. 

### 2.5. Cell Viability Assay

Cells were seeded into 96-well, white clear-bottom plates and allowed to grow under specified conditions. Viability was assessed with CellTiter-Glo luminescent reagent (Promega, Madison, WI, USA) according to manufacturer’s instructions on a SpectraMax iD3 plate reader.

### 2.6. Flow Cytometry

Cells were grown for 5–7 days in 3-D conditions. Spheroids were prepared into single cell suspensions using Cellstripper (Corning, Corning, NY, USA) and needle dissociation. Fluorescent-conjugated antibodies were purchased from Miltenyi Biotec CD44-FITC, CD117-APC, CD133-FITC, and CD133-APC (Table S2). ALDH activity was assessed using the AldeRed Kit (Millipore Sigma, Burlington, MA, USA) and AldeFluor Kit (Stem Cell Technologies, Vancouver, BC, Canada), according to manufacturer’s instructions. Sorting of positive and negative cells was performed on a BDFACS Melody cell sorter, directly into RNA lysis buffer and immediately processed for qRT-PCR studies. Data were analyzed using FlowJo 10.7.1 software.

### 2.7. Animal Experiments

All animal studies were approved by the SDSU Animal Care and Use Committee (protocol approval number-18-04-006H). For xenografts, 500,000 to 500 ACI23 cells in 200 μL of 1:1 Matrigel and PBS were subcutaneously injected into the left flank of 8-week-old female athymic Nu/Nu mice. Mice were weighed and tumors measured twice weekly. Mice were sacrificed once tumors reached 20 mm length or 120 days growth. To investigate tumor-initiation, the number of days for palpable tumors to appear (time to tumor-initiation) was averaged and separated into Early and Late groups.

### 2.8. Immunohistochemistry (IHC)

Tumors were resected from three mice per group, fixed in 10% neutral buffered formalin, and stored in 70% ethanol before processing. Tumors were embedded in paraffin and sectioned at 5um. Antigen retrieval was performed in citrate buffer and quenched with hydrogen peroxide. Slides were incubated with primary antibodies from Cell Signaling Technology (Danvers, MA, USA) (Table S2) followed by an HRP-linked secondary and processed using (3,3’-diaminobenzidine) DAB kit from Vector Laboratories (Burlingame, CA, USA). Four randomly selected images per slide were acquired with a Zeiss Primo Star HAL/LED Microscope and imaged using Toupview. A digital quantification of DAB staining was performed using ImageJ with a FIJI deconvolution package as described previously [22]. Additionally, images were evaluated by three blinded investigators who scored images based on intensity as described [23]. 

### 2.9. Public Database Analysis

The expression of SOX2, OCT4 (POU5F1), and NANOG was compared to disease recurrence of Ovarian Serous Cystadenocarcinoma in TCGA. Z-score was associated with survival outcome by accessing data on the Cancer Genomics Portal Website [24,25,26] and reconstructing in excel by unique patient ID. Of the 579 total cases, 295 had mRNA expression z-scores (relative to all cells “log RNA Seq V2 RSEM”) and 137 were coded for recurrent (dead and alive) and disease free alive.

### 2.10. Statistical Analysis

Statistics were generated using Prism 8.4.3 with data acquired from at least three independent biological replicates. Results are presented as mean +/- SEM. Significance was calculated using either student’s t-test for comparisons of two means or ANOVA for comparisons of three or more means with post hoc test to identify differences between groups as described in figure legends. Differences between means are considered statistically significant at the 95% level (*p* < 0.05). 

### 2.11. Data Availability

RNA sequencing data are available at the NCBI Gene Expression Omnibus under accession number GSE158949.

## 3. Results

**Figure 1:**
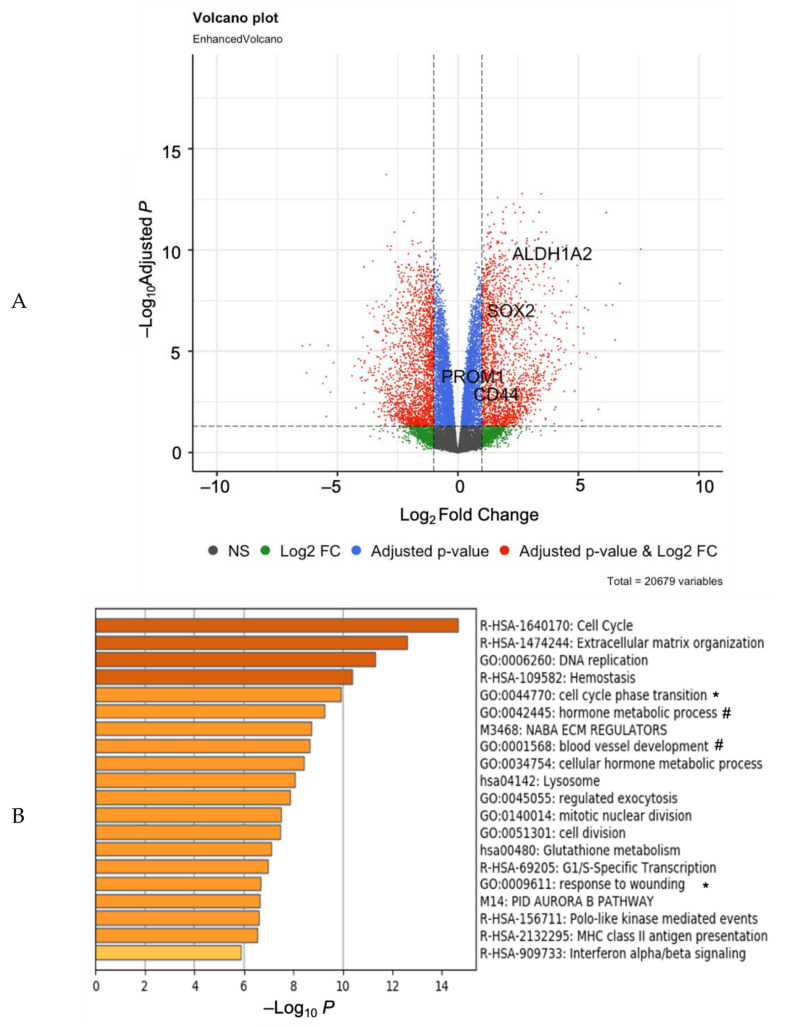

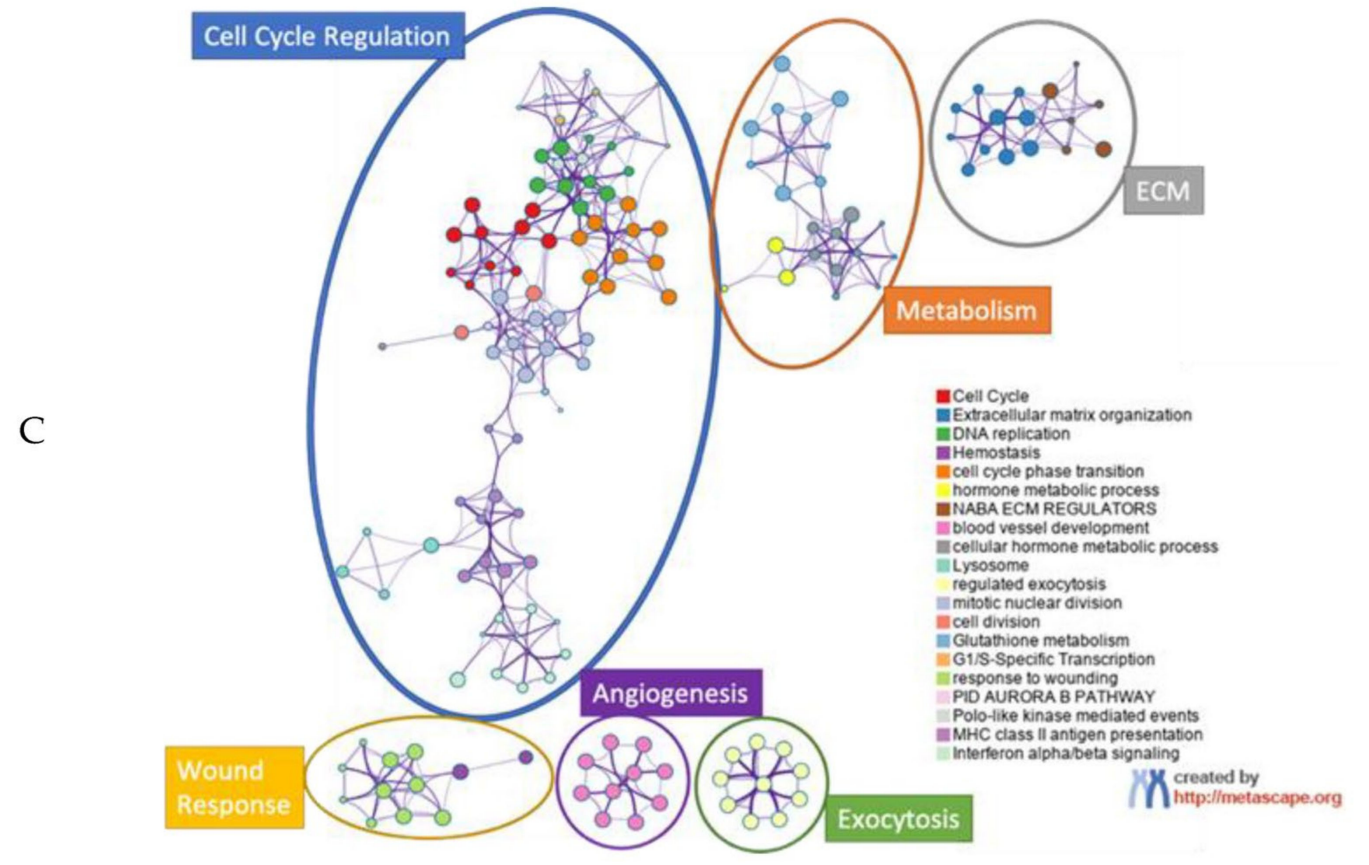
RNA-Sequencing of cells in 3-D relative to 2-D conditions indicate role for *SOX2*. (**A**) Volcano plot of RNA sequencing data showing up- and down- differentially expressed genes (DEGs); Blue genes adjusted *p*-value < 0.05, red genes Log_2_FC< −1 or Log_2_FC > 1 and adjusted *p*-value < 0.05. (**B**) Metascape GO analysis of DEGs Log_2_FC < −1.2 or Log_2_FC > 1.2 and adjusted *p*-value < 0.000001, terms labelled * include *SOX2*, # include *ALDH1A2*. (**C**) Metascape GO tree showing GO clusters.

An analysis of RNA-sequencing data identified 10,222 significantly differentially expressed genes (DEGs) in OV90 cells cultured as spheroids in 3-D conditions, relative to OV90 cells cultured as a monolayer in 2-D conditions (Figure 1A, GEO accession number GSE158949). DEGs representing a twofold change (4045 genes) are indicated in red in the volcano plot and include increased *SOX2* and *ALDH1A2*. DEGs representing fold changes of less than two are indicated in blue and include increased *CD44* and *CD133* (*Prom1*). The higher expression of these genes in 3-D relative to 2-D indicates an enrichment of ovarian TICs, in alignment with our previous findings [9,13,19]. Interestingly, there was no significant difference in the expression of *OCT4*, *NANOG, CD117*, *ALDH1A1,* or other markers of ovarian TICs.

To identify pathways supporting 3-D growth, the top 10% DEGs were selected for gene ontology (GO) studies and subjected to Metascape gene annotation. Metascape analysis indicated that several cell growth processes are altered in 3-D conditions, including cell cycle, extracellular matrix organization, DNA replication, cell cycle phase transition, and wound healing, among others (Figure 1B). The *SOX2* gene (indicated with an asterisk) appears in cell cycle phase transition and wound healing, while *ALDH1A2* gene (indicated with a hash sign) appears in blood vessel development and metabolic process pathways. A Metascape GO tree showed that cell cycle regulation terms cluster together, while metabolism regulation terms also cluster (Figure 1C). These data suggest that in addition to altered metabolism and oxidative stress, which we have previously shown support ovarian cancer spheroids [27], cell cycle regulation plays a critical role in growth in 3-D and may correlate with specific markers of TICs.

**Figure 2:**
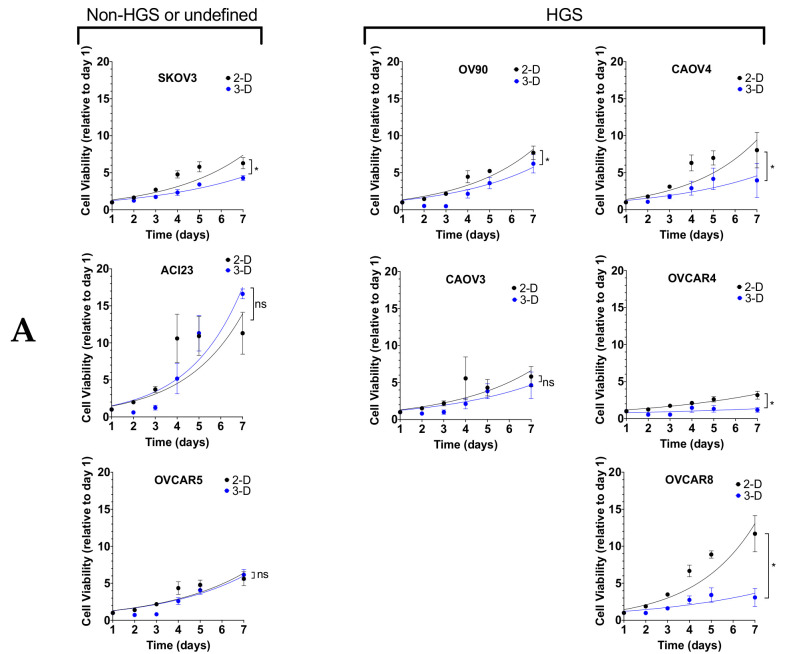

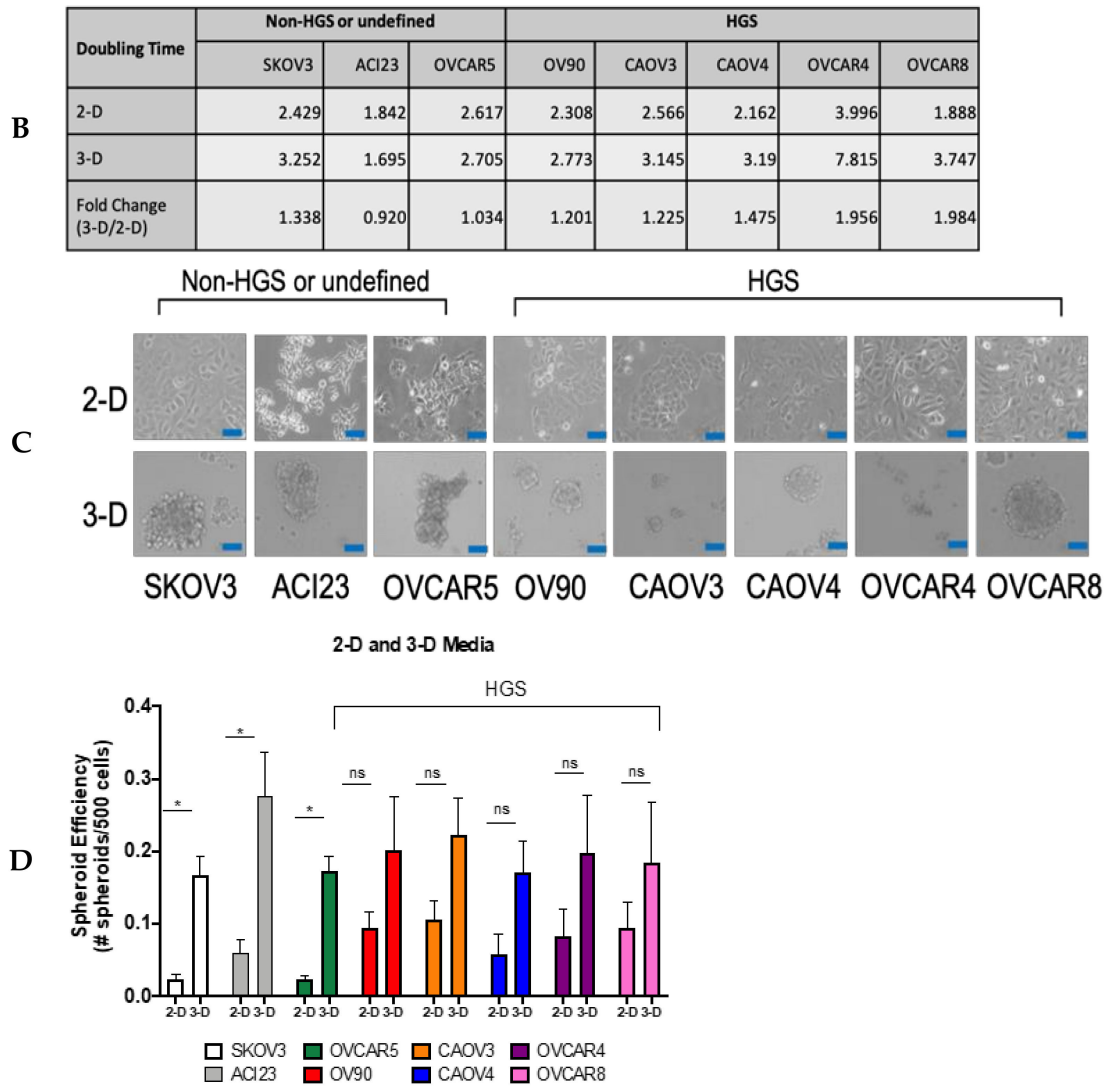
Growth characteristics in 3-D are variable and enhance spheroid formation. (**A**) Cells were seeded in 96 well plates and subjected to Cell-Titer Glo viability assay after 1, 2, 3, 4 and 7 days in culture in 2-D vs 3-D conditions, Two-way ANOVA. (**B**) Doubling time for 2-D and 3-D growth was calculated with Least Squares Fit of Log Exponential Growth. (**C**) Representative brightfield images of ovarian cancer cell lines grown in 2-D or 3-D conditions at 10 × magnification, scale bar 200 µm. (**D**) Spheroid Formation Efficiency for cells grown on ultra-low attachment plates in 3-D media and 2-D media, Student’s T-test 3-D vs. 2-D. Data represent mean and SEM. * *p* < 0.05. ns = not significant.

To investigate the broad applicability of these data, we evaluated a panel of commonly used ovarian cancer cell lines defined by genetic analysis as possibly or likely HGSOC (OV90/CAOV3/CAOV4/OVCAR4/OVCAR8), unlikely HGSOC (SKOV3) [28], or undefined serous (OVCAR5, ACI23) [29,30] (Table S1). Standard 2-D culture conditions revealed differential growth over a seven-day period among the cell lines (Figure 2A). Growth was slower in 3-D conditions for all cell lines except ACI23, which exhibited slightly shorter doubling times, and OVCAR5 and CAOV3, which exhibited no difference or slightly higher doubling times, respectively (Figure 2A,B). ACI23 and OVCAR8 had the shortest doubling time of ~1.8 days each, whereas OVCAR4 had the longest doubling time of ~4 days in 2-D culture (Figure 2B). In accordance with their growth in 2-D, ACI23 cells had the shortest doubling time and OVCAR4 cells had the longest in 3-D culture (Figure 2B). The shorter doubling of ACI23 cells in 3-D relative to 2-D suggests less dependence on serum and anchorage support for growth.

We next measured spheroid formation efficiency in 3-D conditions. Spheroids are multicellular tumor cell aggregates that resemble those found in patient ascites, and are often used as an in vitro surrogate to measure tumor-initiation capacity [9,27,31,32]. All cell lines had relatively equal ability to form spheroids with 3-D media (Figure 2C,D). The high sphere-forming efficiency of ACI23 cells may be attributed to their ability to thrive under serum-deprived, 3-D conditions (Figure 2A). Given the varying response of ovarian cancer cells to growth factor stimulation [33], we also compared spheroid efficiency using standard 2-D media, which contains serum, but lacks EGF and FGF. We found that all cell lines exhibited lower spheroid efficiency when cultured in 2-D media (Figure 2D). The average efficiency across all cell lines in 3-D media was ~0.19, which was reduced to ~0.067 in 2-D media. The SKOV3, ACI23, and OVCAR5 cell lines appear to be most dependent on EGF and FGF, as they had the lowest efficiency in 2-D media (Figure 2D). All cell lines defined as HGSOC exhibited comparable spheroid formation efficiency with either media. 

**Figure 3:**
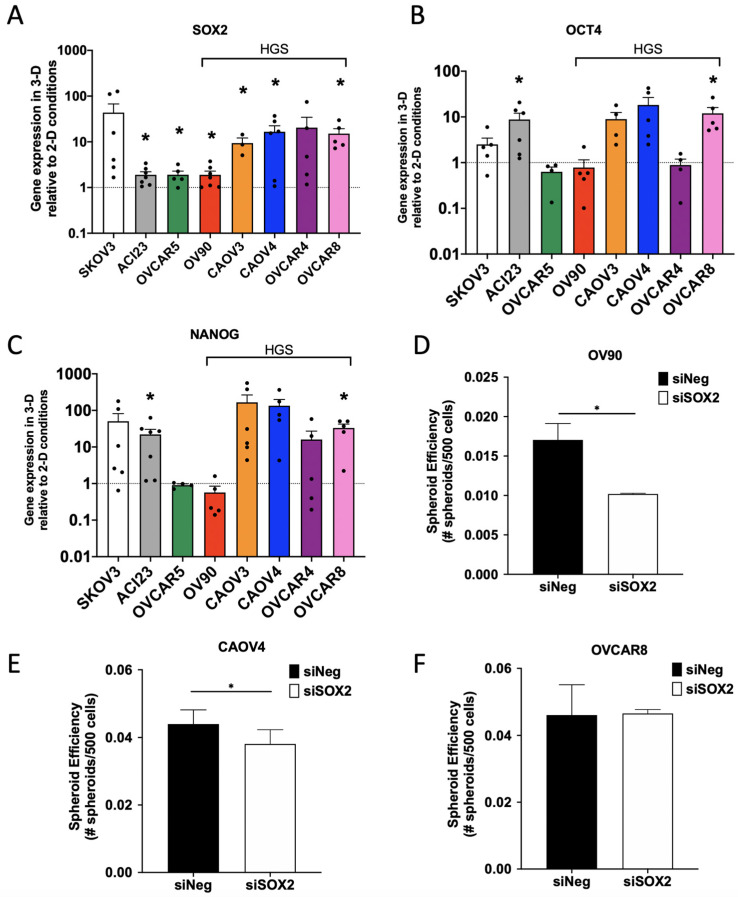
Enrichment of *SOX2* is most consistent across ovarian cancer cell lines and contributes to spheroid formation efficiency. (**A**–**C**) qRT-PCR of selected genes relative to GAPDH housekeeping gene in cells grown for 5 days in 3-D conditions compared to 2-D conditions for (**A**) *SOX2* (**B**) *OCT4* (**C**) *NANOG*, (**D**–**F**) spheroid formation with inhibition of SOX2. Spheroids were measured after growth in 3-D conditions for 4 days after siRNA knockdown of SOX2 in (**D**) OV90 cells, (**E**) CAOV4 cells and (**F**) OVCAR8 cells. Student’s T-test 3-D vs 2-D or siSOX2 vs siNeg. Data represent mean and SEM. * *p* < 0.05.

We next aimed to characterize SOX2, OCT4 and NANOG in our panel and validate the RNA-sequencing data. Although all three embryonic transcription factors are known to support pluripotency and long-term self-renewal, to our knowledge, no broad analysis of their levels in ovarian cancer 3-D cultures has been performed. We first assessed baseline expression of SOX2, OCT4, and NANOG, as well as genes encoding traditional TIC markers in our panel in monolayer, 2-D conditions (Figure S1A–H). As expected, there was heterogeneous expression of TIC genes across the cell lines, with OCT4 and CD44 expressed at similar levels across all lines tested and CD117 having consistently lower expression relative to other markers. We then measured enhancement of gene expression when cultured in 3-D relative to 2-D. All lines showed increased expression of *SOX2* in 3-D relative to 2-D conditions, whereas *OCT4* and *NANOG* enrichment was more variable (Figure 3A–C). The HGSOC lines CAOV3, CAOV4, and OVCAR8, and the SKOV3 and ACI23 lines, had the greatest enrichment of all three genes. Although OV90 is likely HGSOC [28,34], 3-D culture of this line did not enhance the expression of *SOX2*, *OCT4*, and *NANOG* to the same degree as the other HGSOC lines included in this study; however, the significant enrichment of *SOX2* is consistent with the RNA sequencing data provided in Figure 1. Moreover, OV90 cells have elevated endogenous expression of stem cell genes relative to the other HGSOC lines (Figure S1A–H), which may limit any further enrichment in 3-D. To better clarify the role of SOX2 in 3-D growth, we used siRNA to knock down *SOX2* in three representative HGSOC lines (OV90, CAOV4, and OVCAR8) (Figure S2) and assessed spheroid formation efficiency. Our data show that SOX2 knockdown leads to a significant inhibition of spheroid formation efficiency in OV90 and CAOV4 cells, but not in OVCAR8 cells (Figure 3D–F).

We next quantified the transcript levels of *CD44*, *CD117*, *CD133*, *ALDH1A1*, and *ALDH1A2*, genes encoding traditional ovarian TIC markers. In contrast to the enhanced expression of *SOX2*, *OCT4*, and *NANOG* in 3-D relative to 2-D, enhanced expression of ovarian TIC marker genes is more variable across cell lines (Figure S3A–E). Enrichment of either *ALDH1A1* or *ALDH1A2* was evident in 3-D relative to 2-D (Figure S3C–E). Moreover, unlike *ALDH1A1,* which was consistently enriched across all cell lines, *ALDH1A2* was significantly higher in ACI23, OVCAR5, OV90, and CAOV3 cells cultured in 3-D, supporting the RNA sequencing data provided in Figure 1. Differences in *ALDH1A1* and *ALDH1A2* across cell lines may indicate cell-line specific dependence on distinct ALDH isoforms [14,35]. Taken together, these data highlight the heterogeneous expression of TIC genes across ovarian cancer cell lines and suggest that HGSOC lines have consistent enrichment of *SOX2*, *OCT4* and *NANOG* that correlates with specific TIC markers.

**Figure 4:**
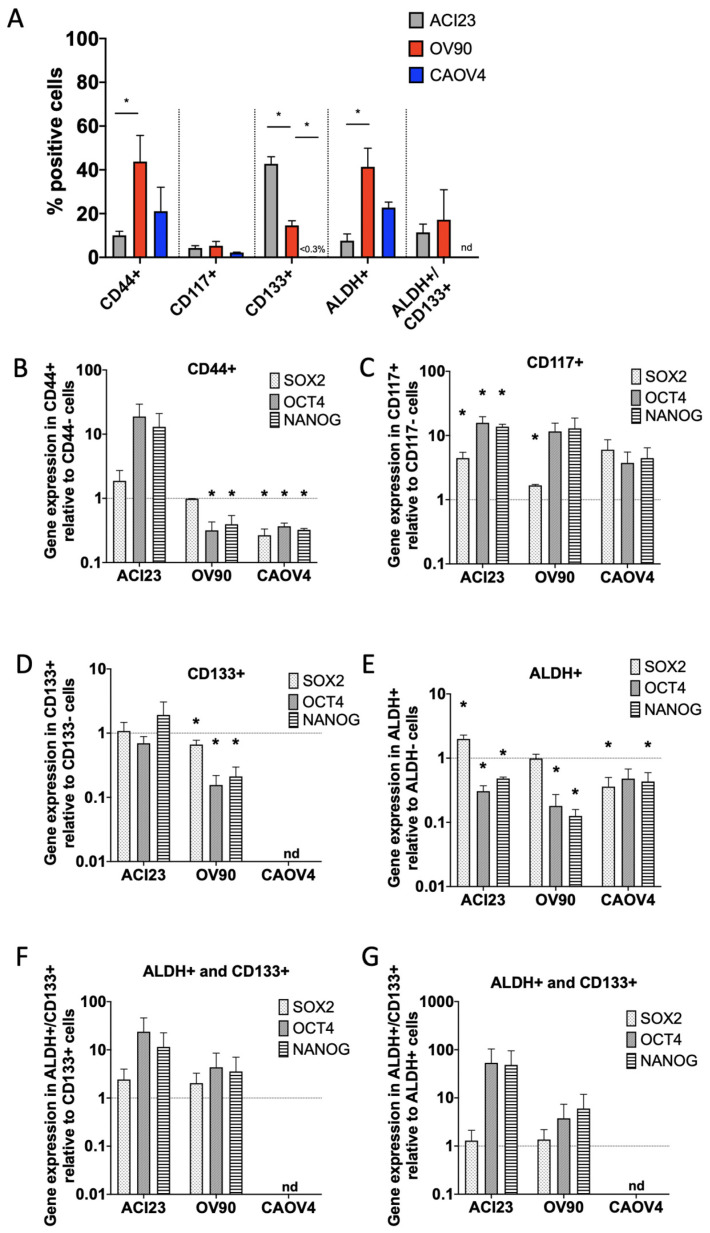
CD117+ and ALDH+/CD133+ cells have elevated *SOX2*, *OCT4* and *NANOG* expression. (**A**) Percent positive cells retrieved from cells grown in 3-D conditions for 5 days and sorted using common stem cell markers (ALDH activity or CD133, CD44, CD117 expression, or ALDH activity and CD133 expression), Student’s T-test. B–G) qRT-PCR of *SOX2*, *OCT4* and *NANOG* in (**B**) CD44+ relative to CD44- cells, (**C**) CD117+ relative to CD117-cells, (**D**) CD133+ relative to CD133- cells, (**E**) ALDH+ relative to ALDH- cells, and ALDH+/CD133+ relative to (**F**) ALDH+ or (**G**) CD133+ cells (OV90 *n* = 2), Student’s T-test positive sorted cells vs negative sorted cells. Data represent mean and SEM relative to control. * *p* < 0.05. nd = not detected.

Although TICs are enriched in 3-D conditions, they remain a minority of the total population [15]. We therefore wanted to establish expression levels of *SOX2*, *OCT4*, and *NANOG* in TICs isolated from 3-D cultures via FACS sorting with traditional TIC markers. We chose to evaluate three lines from our panel: CAOV4 as representative HGSOC, ACI23 as an undefined serous line with exceptional growth in 3-D, and OV90, a likely HGSOC whose growth characteristics and *SOX2*, *OCT4* and *NANOG* in 3-D are distinguishable from the other HGSOC lines in our panel. Each line was sorted for high CD44, CD117, CD133 expression and/or ALDH activity (Figure 4A and Figure S4A–E). The percentage of CD44+ cells was significantly higher in OV90 relative to ACI23 and CAOV4 cells. The percentages of CD117+ cells were less than 10% and not significantly different among the lines. ACI23 and OV90 cells had a significantly higher percentage of CD133+ cells relative to CAOV4, which had less than three percent. Similar to CD44, ALDH was highest in OV90, followed by CAOV4, and lowest in ACI23. Similar to CD117, the percentage of cells which were double positive for ALDH and CD133, a commonly used combination to identify ovarian TICs, was relatively low.

Quantification of *SOX2*, *OCT4*, and *NANOG* in FACS sorted marker positive populations relative to marker negative populations revealed that CD44+ cells had decreased expression of these genes, except in the ACI23 line, which had increased *OCT4* and *NANOG* (Figure 4B). CD117+ cells derived from all three cell lines had enriched *SOX2*, *OCT4*, and *NANOG* (Figure 4C). In contrast, CD133+ cells had no enrichment of *SOX2*, *OCT4* or *NANOG* (Figure 4D). CAOV4 cells had a negligible number of CD133+ cells, and thus, could not be analyzed. Similarly, ALDH+ cells had decreased *SOX2*, *OCT4*, or *NANOG*, except for ACI23 cells, which had higher *SOX2* (Figure 4E). Given the reliability of marker combinations to identify TICs, we then queried gene levels in cells expressing both ALDH and CD133, and found that *SOX2*, *OCT4*, and *NANOG* were higher in the double positive population relative to single positive for either marker, although the differences did not reach statistical significance (Figure 4F,G).

**Figure 5:**
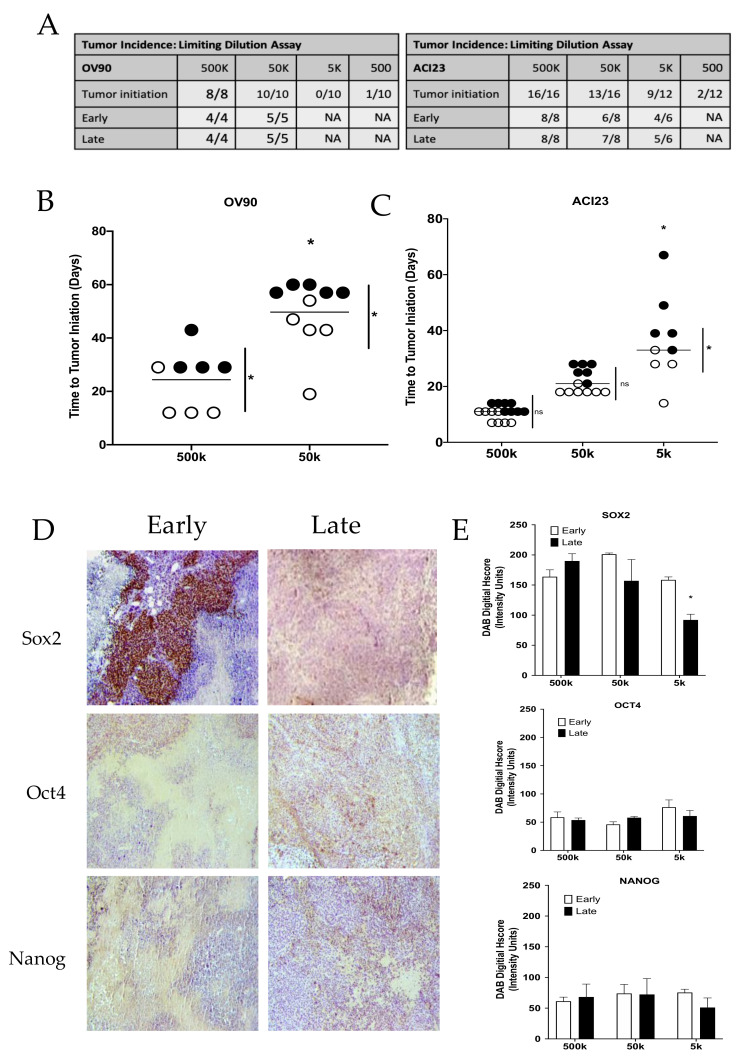
SOX2 expression correlates with tumor-initiation capacity (**A**) Limiting dilution assay comparing early and late appearance of subcutaneous xenograft tumors created with OV90 or ACI23 cells. (**B**–**C**) Time to tumor-initiation is dependent on cell dilution, One-way ANOVA, Tukey Post-hoc test. Early (open circles) and late (closed circles) appearing tumors were significantly different, Two-way ANOVA, Bonferroni post-hoc test. (**D**) Representative images of palpable ACI23 tumors that were resected, fixed, and histologically stained for SOX2, OCT4 or NANOG. (**E**) SOX2, OCT4 and NANOG staining was quantified from three mice from each group using ImageJ and four technical replicates to calculate digital histology score (Hscore), Two-way ANOVA, Bonferroni post-hoc test. Data represent mean and SEM. * *p* < 0.05.

To investigate growth properties in vivo, we injected different dilutions of cancer cells to measure tumorigenicity and the corresponding endogenous expression of SOX2, *OCT4*, and *NANOG*. We chose to evaluate the ACI23 and OV90 lines, since both had consistent enrichment of cells expressing TIC markers (see Figure 4A). Mice that received 500 k or 50 k OV90 cells developed tumors with 100% efficiency, whereas mice that received 5 k or 0.5 k OV90 cells exhibited 0% or 10% efficiency, respectively (Figure 5A). Mice that received 500 k ACI23 cells developed tumors with 100% efficiency, whereas mice that received 50 k, 5 k or 0.5 k ACI23 cells developed tumors with 81.25%, 75%, or 17% efficiency, respectively, over a period of 120 days (Figure 5A). Mice receiving OV90 cells developed palpable tumors (time to tumor-initiation) over a broad range of time with either dilution (Figure 5B). Palpable tumors were evident in 9–13 or 18–25 days in mice that received 500 k or 50 k ACI23 cells, respectively, whereas tumors developed over a broader range of time in mice that received 5k cells (as early as 26 or as late as 45 days) (Figure 5C). We were interested in determining whether this difference in time to tumor-initiation within different dilution groups could be correlated with SOX2, OCT4, or NANOG expression. We first segregated tumors into two groups, early and late, as noted in Figure 5B,C by open or closed circles, and analyzed the differences in time to tumor-initiation. As expected, there were no significant differences in time to tumor-initiation in early and late tumors from 500k or 50k dilution of ACI23 cells (Figure 5C). There were significant differences, however, in time to tumor-initiation in early and late tumors in the 5k dilution of ACI23 cells and in both dilutions of OV90 cells (Figure 5B). Given the fundamental role of SOX2, OCT4, and NANOG in supporting self-renewal properties, we examined their levels in tumors that appeared early, demonstrating enhanced tumor-initiation ability, versus those that appeared late, showing poor tumor-initiation ability. Tumors were resected after reaching an average volume of ~900mm^3^ and quantitatively assessed for SOX2, OCT4, and NANOG expression by immunohistochemical staining (Figure 5D). SOX2 was not significantly different in tumors that developed from 500 k or 50 k dilutions of ACI23 cells; however, it was significantly increased in early appearing tumors that developed from the 5k dilution (Figure 5E and Figure S6). There was no significant difference in OCT4 or NANOG in early or late appearing tumors from any of the dilutions of ACI23 cells. These data suggest that SOX2, relative to OCT4 or NANOG, is more strongly associated with tumor-initiation efficiency, a fundamental feature of TICs. 

**Figure 6:**
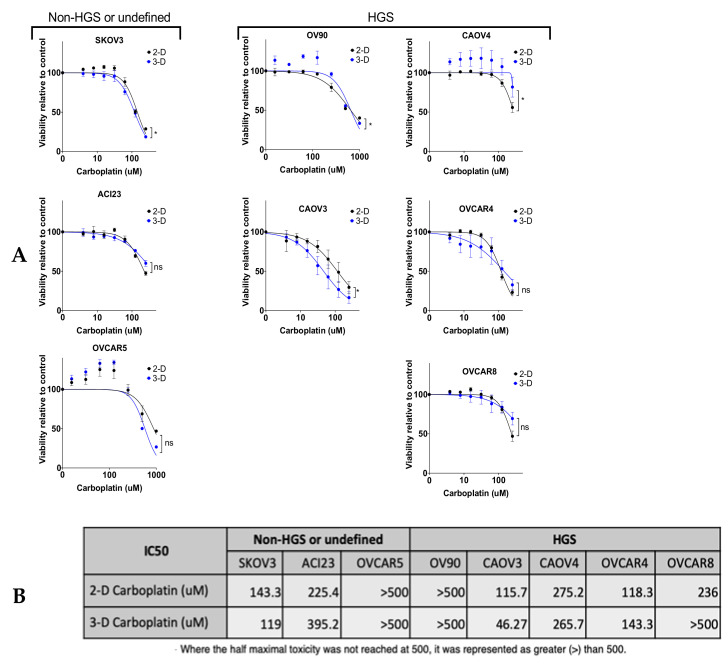

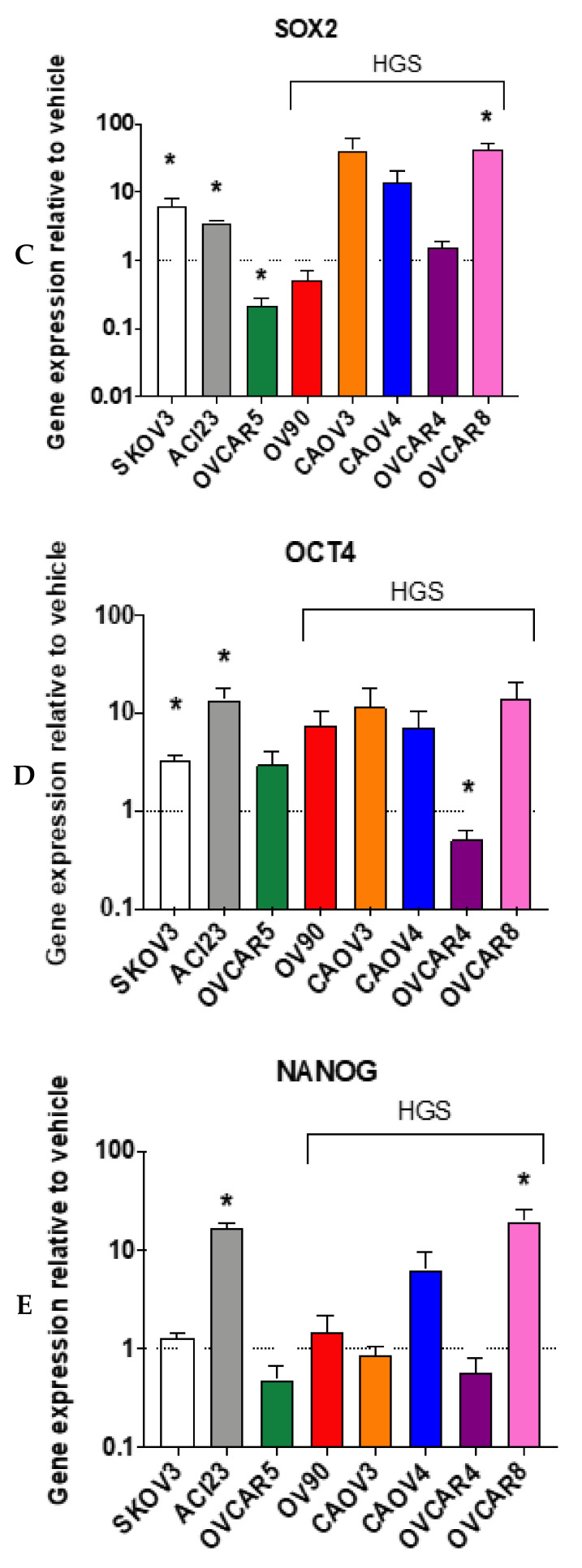

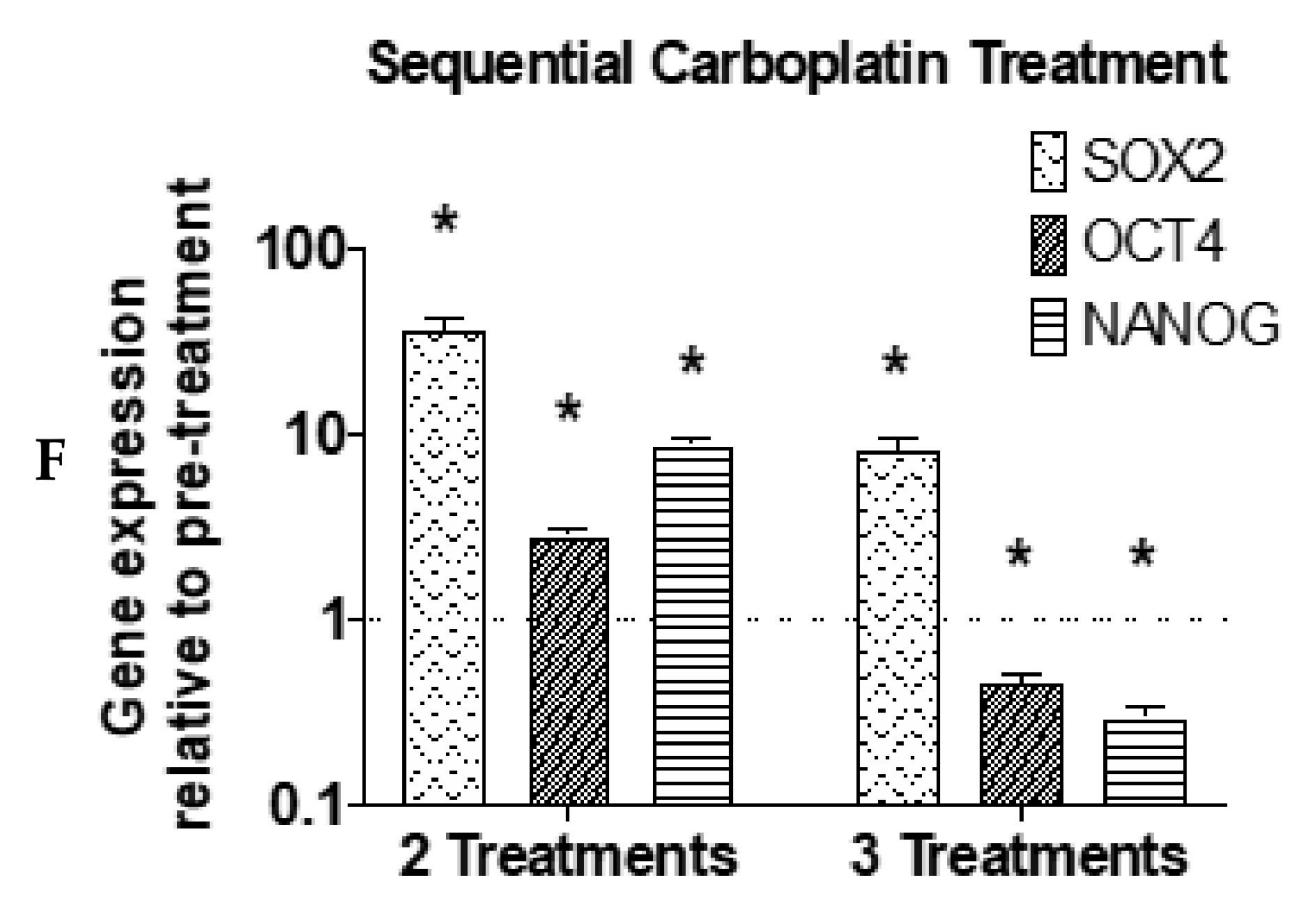
*SOX2* is highly expressed in chemoresistant cells. (**A**) Viability measured after 48-h exposure to a range of concentrations of carboplatin compared to vehicle control for 2-D conditions and 3-D conditions, Two-way ANOVA treated vs vehicle. (**B**) IC_50_ calculations for carboplatin in 2-D and 3-D conditions, calculated with Least Squares Fit. C–E) Cells treated with carboplatin for 72 h at IC_30_ were subjected to qRT-PCR to measure expression relative to vehicle control of (**C**) *SOX2* (**D**) *OCT4* and (**E**) *NANOG*, Student’s T-test treated vs vehicle. (**F**) qRT-PCR of *SOX2*, *OCT4* and *NANOG* genes after sequential carboplatin treatments relative to pre-treatment, Students T-test treated vs pre-treatment. Data represent mean and SEM. * *p* < 0.05.

**Figure 7:**
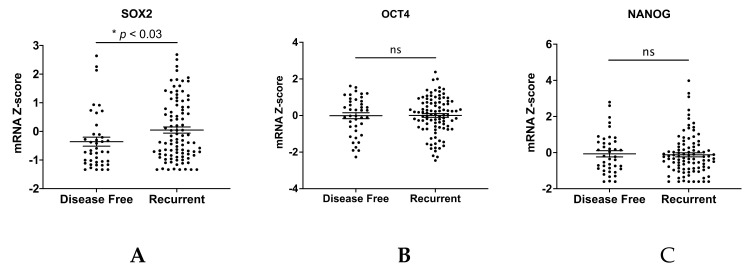
*SOX2* is a marker of recurrence. (**A**–**C**) mRNA Z-score and disease status of patients from HGSOC TCGA data of recurrent cases (*n* = 96) and disease-free cases (*n* = 41) for (**A**) *SOX2*, (**B**) *OCT4*, and (**C**) *NANOG*, Student’s T-test.

We next characterized *SOX2*, *OCT4*, and *NANOG* in the cell line panel after in vitro exposure to carboplatin, another condition that enriches for TICs. We first measured viability in 2-D and 3-D after 48- or 72-h exposure to a range of carboplatin concentrations to generate response curves (Figure 6A and Figure S5A,B). As expected, the cell lines had variable sensitivities to carboplatin, with most lines having higher IC_50_ in 3-D (Figure 6B). We treated 2-D cultures with IC_30_ concentrations of carboplatin for 72 h and examined *SOX2*, *OCT4*, and *NANOG*. Relative to vehicle treated cultures, all cell lines except OVCAR4 had enhanced expression of at least one of the genes (Figure 6C–E). ACI23, CAOV4, and OVCAR8 had elevated expression of all three genes after carboplatin treatment. Given the strong association of SOX2 with spheroid formation and tumor initiation, we evaluated whether knockdown of SOX2 (Figure S2) enhanced the sensitivity of HGSOC lines to carboplatin. Knockdown of SOX2 led to a significant increase in the sensitivity of CAOV4 and OVCAR8 cells to 72-h exposure to carboplatin, while there was no effect seen in OV90 cells (Figure S5C). This may be explained by the fact that CAOV4 and OVCAR8 have enhanced SOX2 expression after carboplatin exposure relative to OV90 cells (Figure 6C). In order to more closely mimic clinical regimens and reproduce biological half-life, we extended these studies in 2-D cultured ACI23 cells that received three sequential treatments of carboplatin. Initial treatments were average maximum serum concentration (38 µM) for 12 h with subsequent two-fold dilution with media (Figure 6F). After two treatments with carboplatin, *SOX2*, *OCT4*, and *NANOG* remained significantly higher relative to pretreatment levels; however, after three treatments, only *SOX2* remained high, while *OCT4* and *NANOG* levels decreased. Taken together, these data suggest a stronger role for SOX2 in chemotherapy resistance, a central feature of TICs that may contribute to cancer recurrence. These findings are supported by TCGA data showing that ovarian cancer cases classified as recurrent (*n* = 96) had significant enrichment of SOX2, but not OCT4 or NANOG, relative to disease-free cases (*n* = 41) (Figure 7A–C) [24,25,36].

## 4. Discussion

Our previous work showed that ovarian cancer cells cultured in 3-D conditions enhanced the growth of multicellular spheroids, enriched for TICs expressing stem cell markers, increased tumor-initiating capacity, and enhanced resistance to chemotherapies [9,21]. We further found that altered drug metabolism and oxidative stress pathways enhanced the growth of ovarian cancer spheroids exhibiting drug resistance and potential for relapse [27]. In this study, we aimed to clarify the role of cell cycle pathway genes, including *SOX2*, that also support the growth of 3-D spheroids and may contribute to relapse. *SOX2*, *OCT4*, and *NANOG* encode master embryonic transcription factors that are vital for quiescence, pluripotency, and long-term self-renewal [18,37], properties which are characteristic of stem-like behavior that may more reliably identify TICs [38]. The expression of these genes has not been fully established in ovarian cancer TICs, so we took a comprehensive approach to characterize their expression in a panel of ovarian cancer cells cultured under different growth and treatment conditions. We also wanted to establish any correlations with traditional surface proteins and ALDH, markers commonly used to isolate TICs. Given the diversity of markers used across current studies, we reasoned that these data may provide a more reliable and functionally relevant indicator of TICs with stem-like properties. 

In order to account for the heterogeneity of ovarian cancer cells [11,33,39,40], we evaluated a panel of commonly reported lines cultured in 2-D and 3-D conditions. We found that although *SOX2*, *OCT4*, and *NANOG* were increased in 3-D conditions relative to 2-D conditions, *SOX2* was the most consistently elevated gene. CAOV3 had one of the highest enrichments of *SOX2* in 3-D growth, in agreement with previous studies of CAOV3 spheroids [13]. Moreover, *SOX2* reached significance across nearly all HGSOC lines, while *OCT4* and *NANOG* expression were variable. Knockdown of *SOX2* led to a significant decrease in spheroid formation efficiency in two out of the three cell lines tested, which is in line with other studies showing that knockdown of SOX2 reduces sphere formation in OVCAR3 cells and that overexpression increases sphere formation in cell lines and patient samples [13]. In contrast to *SOX2*, genes encoding traditional TIC markers were less consistently enriched, except in CAOV3 cells, which exhibited high expression of all genes tested. Interestingly, the *ALDH1A2* gene, unlike the other markers, was enhanced in 3-D cultures relative to 2-D cultures of OVCAR5 and OV90 lines and *ALDH1A1* was enhanced in all lines tested. This may be explained by the dependence of certain cells on different ALDH enzymes, a family comprised of 19 isozymes [35], and the corresponding differences in the endogenous expression of these genes across ovarian cancer cell lines shown by this study and others [14,41]. These data are in agreement with our previous work showing that ALDH1A2, relative to ALDH1A1, was more enriched in OV90 TICs and contributed to spheroid formation [21]. Recent findings demonstrating that ALDH1A2 [41] suppresses the proliferation of ovarian cancer cells may highlight its role in maintaining quiescence of the TIC population, as suggested by our studies. 

In addition to characterizing ovarian TIC gene expression, our work confirms the heterogeneity of ovarian TIC markers [7,15,16,42] and supplements previous findings demonstrating differential expression of CD44, CD117, CD133, and ALDH in the OVCAR5, SKOV3, OV90, and A2780 cell lines [11,43,44]. Due to their increased quiescent nature, we expect TIC populations to represent a minority of the total population. CD117 single positive cells were a minority population of cells across all cell lines tested, in agreement with a previous study of ovarian cancer xenografts showing that CD117+ cells comprised less than two percent of the total tumor cells, and that transplantation assays required 100-fold fewer CD117+ cells relative to CD117- cells [45]. CD133, together with ALDH, is a commonly used marker combination that identifies a minority population of ovarian cancer cells [10,12]; indeed, we found that this combination identified a smaller population relative to either marker alone.

In marker sorted populations, the CD117+ cells or the ALDH+/CD133+ double positive cells had enriched *SOX2*, *OCT4*, and *NANOG* relative to their negative counterparts. Although the FACS sorting studies were completed on only three cell lines, our results suggest that *SOX2*, *OCT4*, and *NANOG*, while being enriched in 3-D cultures, do not necessarily correlate with TIC marker expression at the protein level, and/or their expression is limited to a minor population of CD117+ cells or ALDH+/CD133+ double positive cells [10]. This complements previous studies demonstrating high tumor-initiation capacity of CD117+ or ALDH+/CD133+ cells [10,12,45]. Moreover, these expression patterns exist on a minority of cells in all three lines we tested. CD117 is often used in combination with other markers such as CD133 or CD44 to isolate ovarian TICs [46,47]; however, our data suggest that CD117 alone may be a sufficient or potentially more reliable indicator of TICs with stemness properties associated with SOX2, OCT4 and NANOG. As it identifies a more minor population, CD133 in combination with ALDH identified cells with higher SOX2, OCT4, or NANOG activity relative to either marker alone. Another study showed differential endogenous expression of *SOX2*, *OCT4*, and *NANOG* in OVCAR5 cells that was enhanced only in cells sorted for CD133 in combination with CXCR4, relative to CD133 alone [48]. 

The high expression of SOX2, relative to that of OCT4 or NANOG, in early appearing tumors, in chemotherapy treated cells, and in recurrent patient samples suggests a stronger role for SOX2 in supporting TIC properties. These findings are consistent with other studies demonstrating that SOX2 is critical for ovarian cancer spheroid formation, tumor-initiation, and worse overall survival [13,19,49,50]. Through its downregulation of cell cycle proteins such as cyclin D1 and CDK4, SOX2 may specify a quiescent phenotype that can resist cytotoxic therapies [51]. Although siRNA knockdown of SOX2 led to a small but significant enhancement of chemosensitivity in two out of three lines tested, we expect that larger differences may occur over longer periods of chemotherapy exposure where TICs are enriched after repeated exposures. In addition to regulating cell cycle progression, SOX2 also is vital for maintaining an undifferentiated phenotype through its regulation of pluripotency and differentiation enhancers [52]. Although OCT4 appears to perform similar functions, it associates at chromosomal regions independent of those occupied by SOX2 [52]. It has also been shown that fluctuations in SOX2 or OCT4 levels may influence cell fate [53]. Presumably, the efficient reestablishment of heterogeneous tumors following chemotherapy would rely on flexible mechanisms that permit chemoresistance, pluripotency, and long-term self-renewal, processes which are largely influenced by SOX2, OCT4, or NANOG. Although additional studies are required to confirm SOX2 as a bona fide TIC marker, our study indicates that SOX2 may be a driver of recurrence which could serve as a more reliable marker of ovarian TICS with high relapse potential.

Over a decade of research has identified TICS as chemoresistant cells that may be responsible for relapse in many cancer types; however, their heterogeneity remains a challenge. In this study, we sought to establish whether SOX2, OCT4, or NANOG are reliable indicators of ovarian TICs with enhanced quiescence and long-term self-renewal. Our in vitro and in vivo studies indicated that SOX2, relative to OCT4 and NANOG, correlates with 3-D spheroid growth, tumor-initiating capacity, and chemoresistance. Finally, TCGA datasets show that *SOX2*, but not *OCT4* or *NANOG*, is significantly elevated in recurrent disease. These data complement current literature describing ovarian TICs and suggest that SOX2 should be considered when evaluating TIC properties in ovarian cancer cells. A better understanding of how SOX2 contributes to TIC biology will lead to more effective therapeutic strategies for preventing relapse and prolonging remission for ovarian cancer patients.

## 5. Conclusions

In this study, we evaluated *SOX2*, *OCT4*, or *NANOG* in ovarian TICs, and used in vitro and in vivo studies to determine that *SOX2*, relative to *OCT4* or NANOG, more strongly correlates with 3-D spheroid growth, tumor-initiating capacity, and chemoresistance in ovarian cancer cells, regardless of histology. Although the expression of TIC markers is heterogeneous among cell lines, a minority population of cells expressing CD117 alone or CD133 together with ALDH have high expression of *SOX2*, *OCT4*, and *NANOG*. Our analysis of TCGA datasets show that the expression of *SOX2*, but not of *OCT4* or *NANOG*, is significantly elevated in recurrent disease. Altogether, our findings suggest that SOX2 should be considered when evaluating ovarian cancer TIC populations and relapse potential. Identifying more reliable markers of TICs is critical for understanding TIC biology and developing therapies to prevent disease recurrence.

## Data Availability

The RNA sequencing data are available at the NCBI Gene Expression Omnibus under accession number GSE158949. The TCGA data is a publicly available dataset and can be found here: [www.cbioportal.org]. Additional data presented in this study are available by request to the corresponding author.

## Supplementary Materials

The following are available online at https://www.mdpi.com/2072-6694/13/2/262/s1, Table S1: Cell Line Information for Ovarian Cancer Panel, Table S2. Reagent and Manufacturer Order Information, Figure S1: Normalized Expression of genes encoding TIC markers, Figure S2: Western blot of SOX2 siRNA knockdown, Figure S3: Expression of genes encoding TIC surface markers, Figure S4: Gating strategy for FACS Experiments, Figure S5: Time Course for in vitro Chemotherapy Treatment Experiments, Figure S6: IHC staining ranks.
