# Supplementary material for: Characterization of SOX2, OCT4 and NANOG in Ovarian Cancer Tumor-Initiating Cells
Source: Cancers (Basel). 2021 Jan 12;13(2):262. doi: 10.3390/cancers13020262 (PMC7828139; doi:10.3390/cancers13020262)
Supplement: Supplementary file 1 [file cancers-13-00262-s001.pdf]

Supplementary Materials

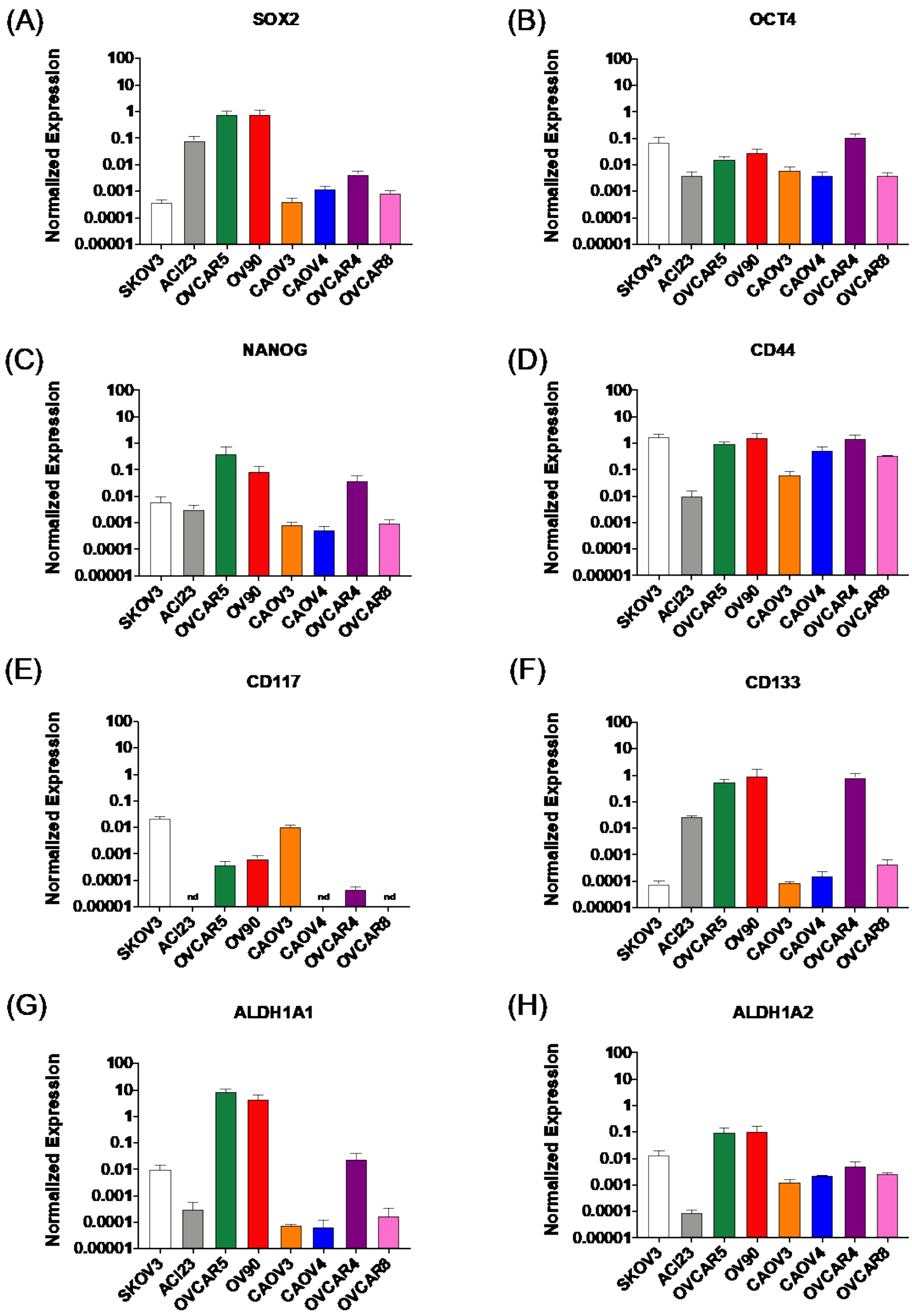

Figure S1. Normalized Expression of genes encoding TIC markers. A-H) qRT-PCR of selected genes relative to GAPDH housekeeping gene in cells grown for 5 days in 2-D conditions for (**A**) SOX2 (**B**) OCT4 (**C**) NANOG (**D**) CD44 (**E**) CD117 (**F**) CD133 (**G**) ALDH1A1 and (**H**) ALDH1A2. Data represent mean and SEM. nd = not detected.

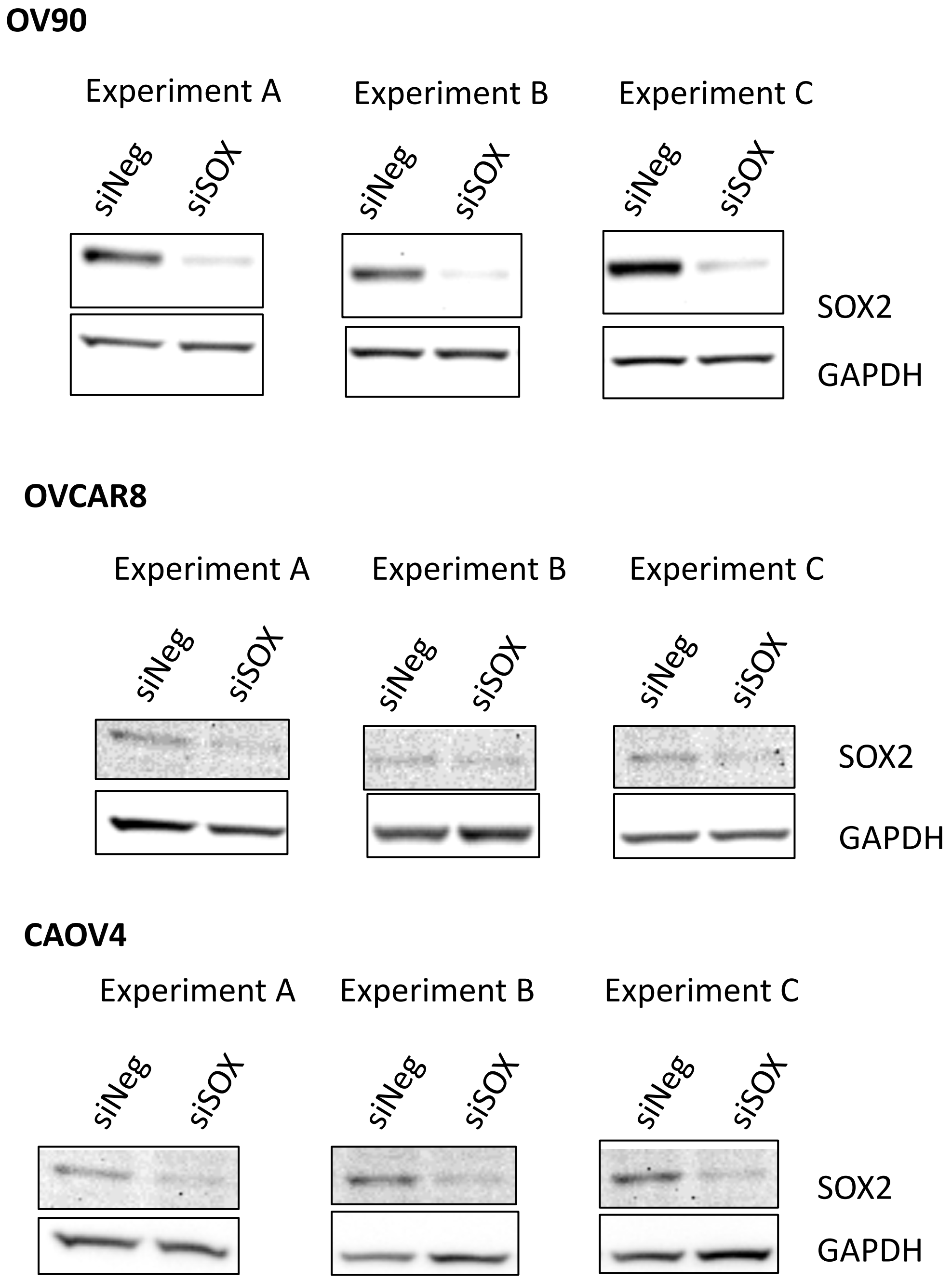

Figure S2. Validation of siRNA knockdown of SOX2. Protein lysates were collected from three biological replicates (Experiments A-C) 72 hours after siRNA transfection and analyzed by western blot for SOX2. Each lane was loaded with 40 mg of protein and membranes blotted with anti-SOX2 antibody overnight. Each experiment demonstrates consistent knockdown of SOX2.

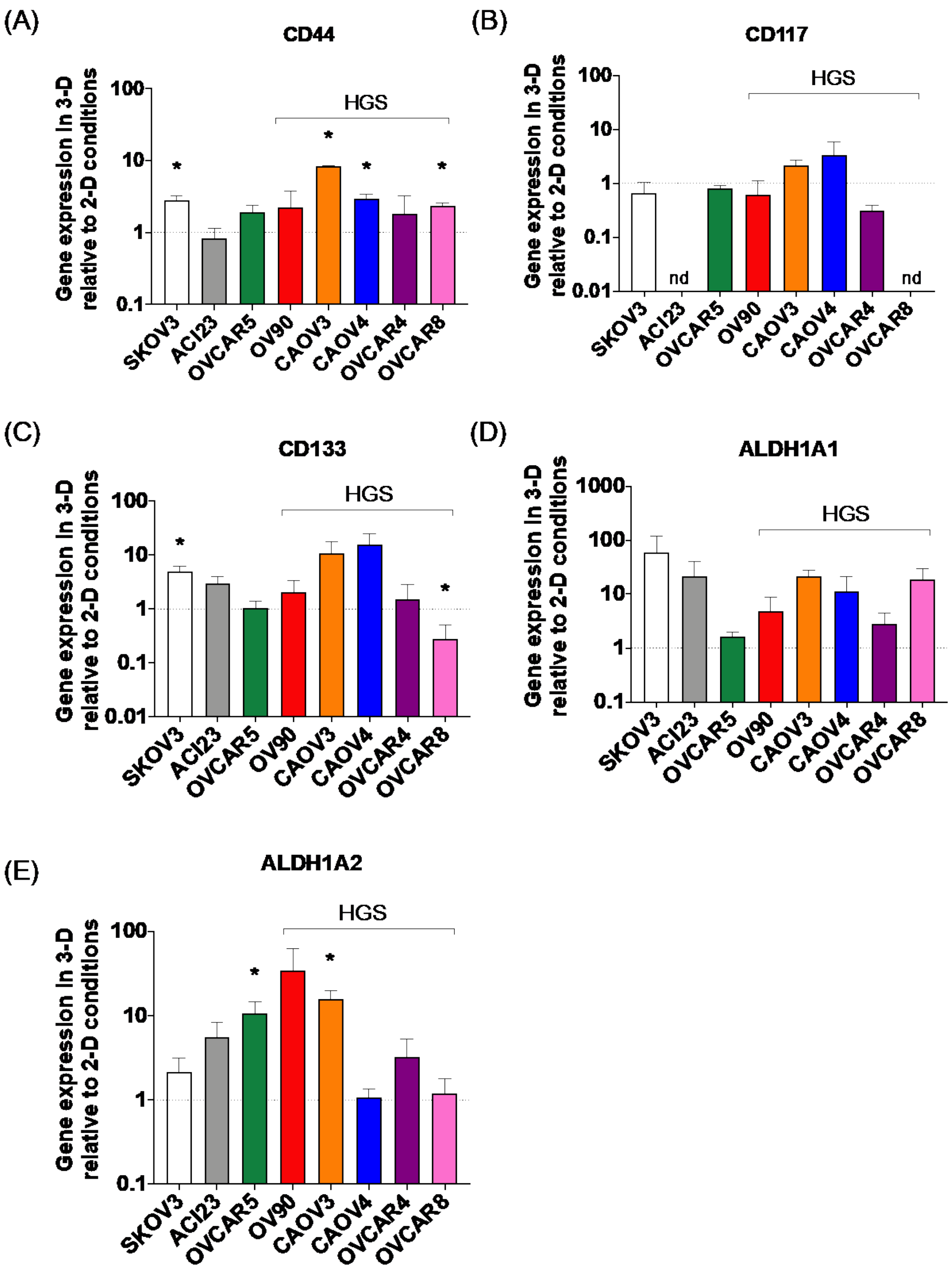

Figure S3. Expression of genes encoding TIC surface markers. qRT-PCR of selected genes relative to GAPDH housekeeping gene in cells grown for 5 days in 3-D conditions compared to 2-D conditions for A) CD44 B) CD117 C) CD133 D) ALDH1A1 and E) ALDH1A2, Students T-test 3-D vs 2-D; Data represent mean and SEM. nd = not detected. * $p < 0.05$.

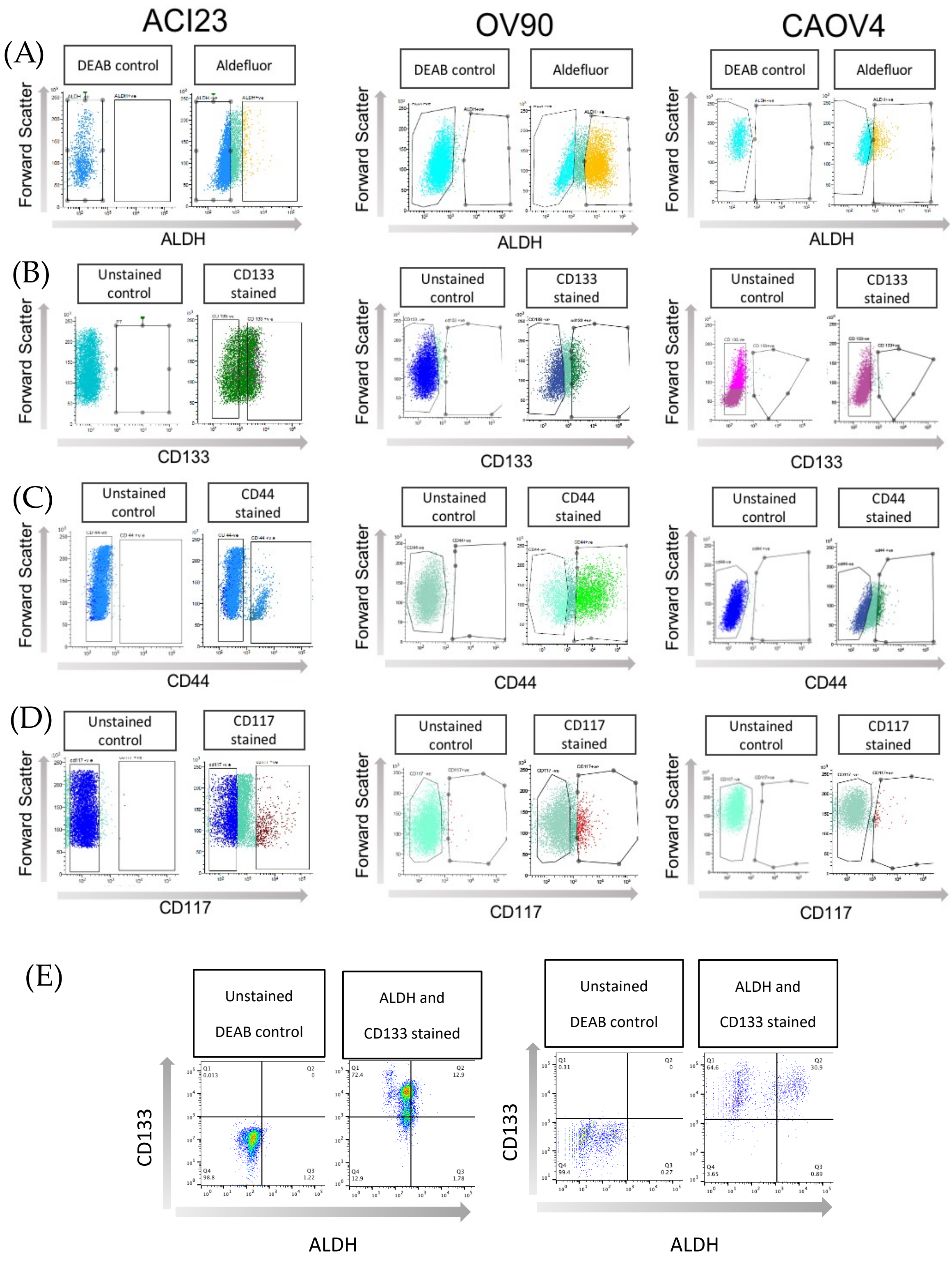

Figure S4. Gating strategy for FACS Experiments. Representative gates for sorting of ACI23, OV90, and CAOV4 ovarian cancer cells with (**A**) high ALDH activity, (**B**) CD133 expression, (**C**) CD44 expression, (**D**) CD117 expression, or (**E**) double stained cells expressing CD133 and high ALDH activity.

(A)

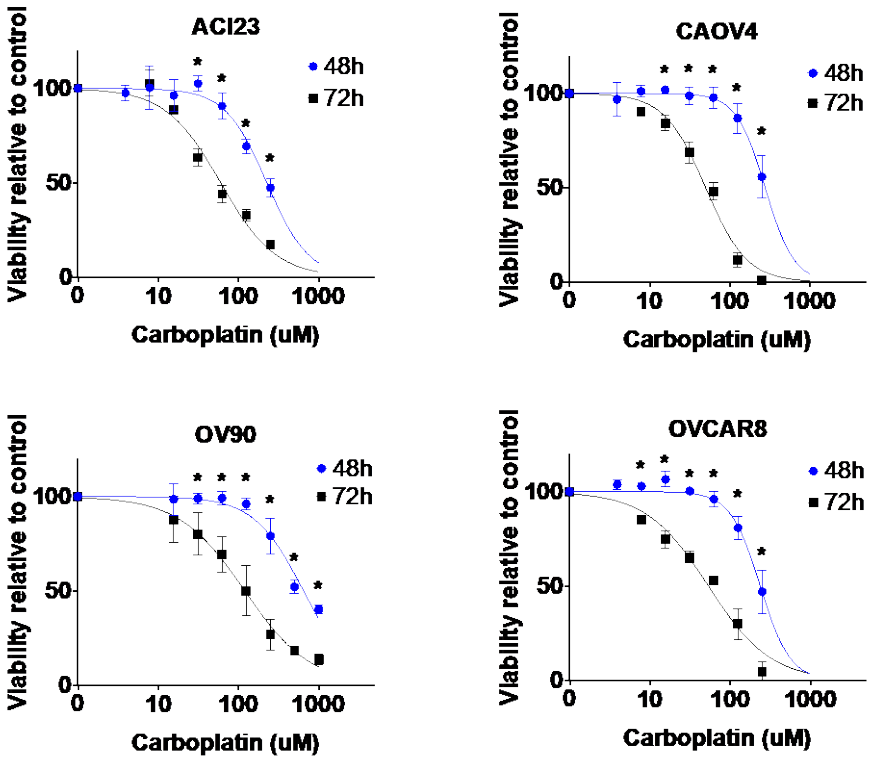

(B)

IC50	Non-HGS or undefined	HGS		
	ACI23	OV90	CAOV4	OVCAR8
48h 2-D Carboplatin (uM)	225.4	647.4	275.2	236.0
72h 2-D Carboplatin (uM)	59.7	119.8	49.9	48.4

(C)

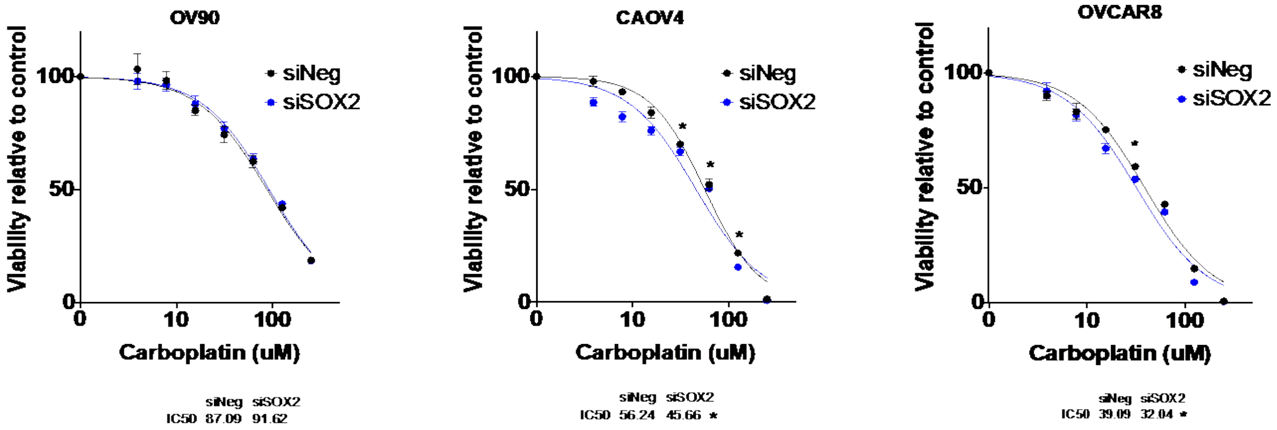

Figure S5. Time Course for in vitro Chemotherapy Treatment Experiments. (**A**) Viability of cells cultured in 2-D for 48 or 72 hours in a range of concentrations of carboplatin compared to vehicle control for ACI23, OV90, CAOV4, and OVCAR8 cells; (**B**) IC_{50} calculations for carboplatin in 2-D conditions for 48 or 72hrs; (**C**) Carboplatin sensitivity after siRNA knockdown of SOX2 and 72 hrs exposure to range of concentrations of carboplatin. Graphs calculated with Least Squares Fit. Two-way ANOVA, Bonferroni post-hoc test Data represent mean and SEM. * $p < 0.05$.

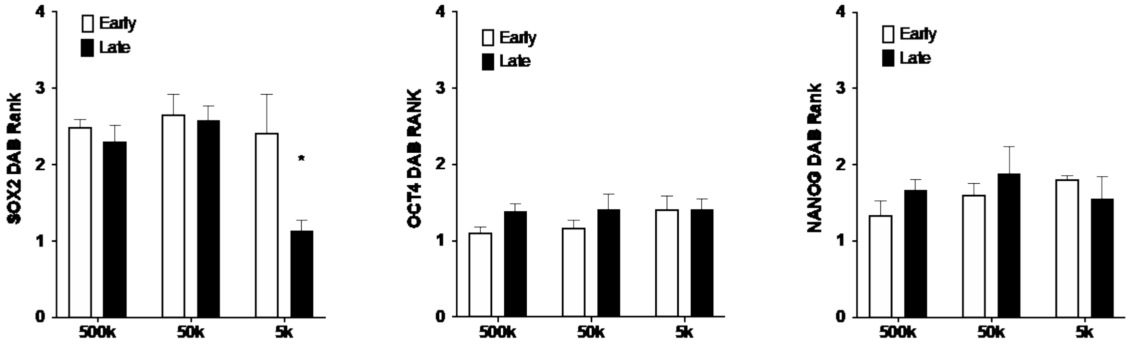

Figure S6. SOX2 expression correlates with tumor-initiation. SOX2, OCT4 and NANOG staining was quantified through ranking of intensity by three blinded investigators, Two-way ANOVA, Bonferroni post-hoc test. Data represent mean and SEM. * $p < 0.05$.

Table S1. Cell Line Information for Ovarian Cancer Panel.

	Non-HGS or Undefined			High Grade Serous (HGS)				
**Cell Lines**	**SKOV3**	**ACI23**	**OVCAR5**	**OV90**	**CAOV3**	**CAOV4**	**OVCAR4**	**OVCAR8**
**Domcke Designation**	Unlikely HGSOC	NA	NA	Possibly HGSOC	Likely HGSOC	Likely HGSOC	Likely HGSOC	Possibly HGSOC
**Histology**	Serous	Serous	Adeno-carcinoma G1	Adeno-carcinoma	Adeno-carcinoma	Adeno-carcinoma	Adeno-carcinoma G2	Adeno-carcinoma G3
**Cell Line Source**	ATCC: HTB-77	J.I Risinger, MSU	NCI-DTP: OVCAR-5	ATCC: CRL-11732	ATCC: HTB-75	ATCC: HTB-76	NCI-DTP: OVCAR-4	NCI-DTP: OVCAR-8
**Maintenance Media (Gibco)**	RPMI	DMEM/F 12	RPMI	RPMI	RPMI	RPMI	RPMI	RPMI
**Patient Source**	Ascites Derived	Primary Site	Ascites Derived	Ascites Derived	Primary Site	Metastatic Site	Ascites Derived	Primary Site

Table S2. Reagent and Manufacturer Order Information.

Reagent (in order of appearance in methods)	Company and Order Information
RPMI 1540 Media	Gibco 11875135
DMEM/F12 Media	Gibco 11320033
KnockOut™ Serum Replacement	Gibco 10828028
Insulin-transferrin-selenium (ITS-G)	Gibco 41400045
Ultra-low attachment flat bottom 96-well plates	Corning 3474
ImageXpress	Molecular Devices ImageXpress Pico
CellReporterXpress Software	Molecular Devices Version 2.1.5156
Hoechst	ThermoFisher Scientific Hoechst 33342
siRNA pools	Dharmacon<br>siSOX2 L-011778-00-0005<br>siNeg D-001810-10-05
Lipofectamine	Lipofectamine RNAiMAX<br>ThermoFisher Scientific 13778075
RIPA lysis buffer	ThermoFisher Scientific 89901
Halt Protease and Phosphatase Inhibitor Single-Use Cocktail	ThermoFisher Scientific 78443
Pierce Rapid Gold BCA Protein Assay Kit	ThermoFisher Scientific: A53227
NuPAGE 4-12% Bis-Tris Protein Gels	ThermoFisher Scientific NP0322BOX
NuPAGE MOPS SDS Running Buffer	ThermoFisher Scientific NP0001
Western Blot Antibodies	SOX2 Primary: Cell Signaling Technology 3579<br>GAPDH Primary: MilliporeSigma MAB374

	Mouse Secondary HRP-linked: Cell Signaling Technology 34577<br><br>Rabbit Secondary HRP-linked: Cell Signaling Technology 7074
SuperSignal Chemiluminescent Substrate	ThermoFisher Scientific SuperSignal West PLUS Chemiluminescent Substrate 34577
iBright Imaging System and Software	ThermoFisher Scientific iBRIGHT CL1000 Imaging System and Analysis Software
NucleoSpin RNA Plus kit	Macherey-Nagel 740984.50
Direct-zol RNA Miniprep Plus Kit	Zymo Research R2071
SpectraMax QuickDrop	Molecular Devices
High-Capacity cDNA Reverse Transcription Kit	ThermoFisher Scientific 4368814
QuantStudio 3 machine and Design software	ThermoFisher Scientific Version 1.5.1
Taqman Probes	ThermoFisher Scientific<br><br>Oct4 Assay ID: Hs04260367_gH<br><br>Sox2 Assay ID: Hs01053049_s1<br><br>Nanog Assay ID: Hs02387400_g1<br><br>GAPDH Assay ID: Hs99999905_m1<br><br>CD44 Assay ID: Hs01075862_m1<br><br>CD117 Assay ID: Hs00174029_m1<br><br>CD133 Assay ID: Hs01009259_m1<br><br>ALDH1A1 Assay ID: Hs00946916_m1<br><br>ALDH1A2 Assay ID: Hs00180254_m1
CellTiter-Glo luminescent reagent	Promega G7570
SpectraMax iD3 plate reader	Molecular Devices

SoftMax Pro Software	Molecular Devices Version 7.1.0
CellStripper	Corning 25-056-CI
Fluorescently-conjugated antibodies from Miltenyi Biotec	CD44-FITC 1:100 (#130-113-341)<br>CD117-APC 1:100 (#130-111-671)<br>CD133-FITC 1:100 (#130-110-968)<br>CD133-APC 1:50 (#130-111-080)
ALDH Activity Assay	AldeRed (Millipore Sigma scr150)<br>AldeFluor (Stem Cell Technologies 01700)
BDFACS Melody cell sorter and BDFACS Chorus Software	Becton Dickinson
FlowJo	Becton Dickinson FlowJo Version 10.7.1
Matrigel	Corning Matrigel 354263<br>Lot number 8260015
IHC Antibodies from Cell Signaling Technology	SOX2 (#3579S)<br>OCT4 (#2750S)<br>NANOG (#4903S)<br>HRP-linked polymer (#8114S)
DAB kit	Vector Laboratories SK-4100
Prism	GraphPad Prism Version 8.4.3
